# Five years’ experience of the clinical exome sequencing in a Spanish single center

**DOI:** 10.1038/s41598-022-23786-6

**Published:** 2022-11-10

**Authors:** A. Arteche-López, A. Ávila-Fernández, R. Riveiro Álvarez, B. Almoguera, A. Bustamante Aragonés, I. Martin-Merida, M. A. López Martínez, A. Giménez Pardo, C. Vélez-Monsalve, J. Gallego Merlo, I. García Vara, F. Blanco-Kelly, S. Tahsin Swafiri, I. Lorda Sánchez, M. J. Trujillo Tiebas, C. Ayuso

**Affiliations:** 1 Department of Genetics, Health Research Institute–Jimenez Diaz Foundation University Hospital (IIS-FJD), Avda. Reyes Católicos 2, 28040 Madrid, Spain; 2 Department of Genetics, University Hospital, 12 de Octubre, Madrid, Spain

**Keywords:** Disease genetics, Genetic testing, Genetics

## Abstract

Nowadays, exome sequencing is a robust and cost-efficient genetic diagnostic tool already implemented in many clinical laboratories. Despite it has undoubtedly improved our diagnostic capacity and has allowed the discovery of many new Mendelian-disease genes, it only provides a molecular diagnosis in up to 25–30% of cases. Here, we comprehensively evaluate the results of a large sample set of 4974 clinical exomes performed in our laboratory over a period of 5 years, showing a global diagnostic rate of 24.62% (1391/4974). For the evaluation we establish different groups of diseases and demonstrate how the diagnostic rate is not only dependent on the analyzed group of diseases (43.12% in ophthalmological cases vs 16.61% in neurological cases) but on the specific disorder (47.49% in retinal dystrophies vs 24.02% in optic atrophy; 18.88% in neuropathies/paraparesias vs 11.43% in dementias). We also detail the most frequent mutated genes within each group of disorders and discuss, on our experience, further investigations and directions needed for the benefit of patients.

## Introduction

Exome sequencing (ES) has revolutionized the diagnosis of genetic disorders, and has contributed to the discovery of new Mendelian-disease genes^1^. Although nearly 85% of the disease causing variants are located in the coding regions or in the canonical splicing sites^2^, a global molecular diagnosis is nowadays only established in up to 25%-30% of cases^3–7^. However, this ES diagnostic capacity is variable and it depends on the analysed disorder^5,7^, the type of study performed (TRIO (31–37%) vs Singleton (21–22%)^6,8^ and the cohort investigated^9,10^.

The clinical exome sequencing -which comprises genes associated to known clinical phenotypes and covers > 98% of variants identified on targeted next-generation sequencing (NGS) panels^11^- has been implemented in many clinical genetics laboratories, becoming a robust and cost-efficient diagnostic tool^12^, despite its inherent challenges and limitations^13^.

A recent overview of the different genetic approaches used to evaluate undiagnosed diseases has been published, emphasizing on how NGS can be incorporated in routine clinical practice^14^. The Genetics Department of the Fundación Jimenez Diaz University Hospital (FJD-HU) in Madrid (Spain) is a well-established and experienced genetics laboratory that integrates cytogenetic and molecular techniques. Before the NGS-era, the analysis of point variants and the sequencing of genes were offered by Sanger sequencing. Gradually, after the NGS implementation, its protocols have been adapted to this new technology. Nowadays, we offer all our patients an initial clinical evaluation and a molecular testing for each disorder being the NGS and, specifically CES, the first diagnostic approach for many of them.

Here, we present a 5-year retrospective review of 4974 clinical exomes performed (from October 2016 to April 2021), analysed and informed in our laboratory. We describe our NGS cases, their distribution, and the global and the specific diagnostic rate (DR) achieved for each type of disorder. We also remark the importance of having an experienced team of clinical and molecular geneticists and emphasise the importance of appropriate communication with fellow clinicians, in an increasingly complex and demanding field.

## Methods

### Patients and samples

All patients were referred to the Genetics Department of the Fundación Jimenez Diaz University Hospital (Madrid, Spain) with a clinical suspicion of a genetic disorder from different Clinical Departments. All underwent full clinical genetic evaluations. Only if indicated and after an adequate pre-test genetic counselling, patients were molecularly studied. Written informed consent was obtained from each participant in accordance with institutional requirements. The study was reviewed and approved by the Research Ethics Committee of HU-FJD and fulfilled the principles of the Declaration of Helsinki.

Whole peripheral blood samples were collected from probands (n = 4974). Data on sex, age and available clinical indication for genetic testing was retrospectively recollected.

Genomic DNA was extracted following standard procedures. From October 2016 to April 2021, clinical exomes were performed using the TruSight One Sequencing panel (TSO; *Illumina In, San Diego, CA, USA*) (N = 612), and the Clinical Exome Solution (CES; *Sophia Genetics*, *USA*) (N = 4362) that capture 4813 and 4900 genes related to inherited diseases, respectively. Over 95% of the captured genes overlapped in both approaches. The detection of Copy Number Variants (CNVs) was only possible in samples sequenced with CES.

All samples were sequenced on a NextSeq500 instrument. Bioinformatics analysis for the detection of single-nucleotide variants (SNVs) and small insertions/deletions (indels) in TSO samples was performed using the Burrows-Wheeler alignment (BWA) Enrichment App, which provides industry-standard alignment. Variant calling was based on the Genome Analysis Toolkit (GATK). An in-house Sophia Genetics pipeline was used for the analysis of single nucleotide variants in CES samples. The detection of CNVs in CES samples was also performed using an in-house Sophia Genetics method based on a hidden Markov model. Specifically, the method evaluates the coverage levels of the target regions across all samples within the same sequencing run. The coverage is normalized by sample and target region, thus enabling CNV calling. A minimum of 8 samples per run is required.

Informed NGS variants in the first 825 clinical exomes of fully characterized cases were validated by Sanger sequencing before reporting^15^. In the remaining samples, only indels and bad-quality NGS variants (filter ≠ PASS, QUAL < 100, coverage < 20X or variant fraction < 20%) were further confirmed.

For CES samples, all CNVs were confirmed before being reported, either by Multiplex ligation-dependent Probe amplification (MLPA) or by array-CGH (60 k), depending on the availability of the MLPA kit and the size of the CNVs.

The analysis of the mitochondrial DNA or the clinical exome analysis in trio was not performed in any of the analysed patients.

### Panel content and variant's interpretation

The Variant Studio (-*Illumina*-) and the SOPHiA DDM™ (*Sophia Genetics*, *USA*) platforms were used for the analysis of variants.

Variant interpretation was performed by our team of experienced molecular geneticists, making use of population and clinical databases (gnomAD, ClinVar), the literature and other tools such as Varsome or InterVar. Variants were classified according to the ACMG criteria in the 5 established categories^16^: benign (B), likely benign (LB), unknown clinical significance (VUS), likely pathogenic (LP) and pathogenic (P). Only P and LP variants, as well as VUS clearly related with the phenotype were reported.

In some cases, when only a LP or P variant was detected in a recessive related-gene, additional testing such as MLPA, array CGH or Sanger sequencing of uncovered regions was performed to attempt detecting the second allele, specifically in samples analysed by TSO as CNVs are not detected with this technology.

Clinical virtual panels were developed based on public database (OMIM, Genomics England PanelApp, DisGeNet, GTR) and medical specific literature. Specifically, retinal dystrophy panels were based on the RetNet database. Each panel was periodically re-evaluated—at least once a year- and thus, the panel content changed over time during the study.

### Data analysis

“Molecular diagnosis” was defined as the presence of a P or LP variant in a dominant or X linked gene; as two P or LP, one P and one LP variant in a recessive gene, respectively, sex regardless. This group was used to define the global DR in our cohort and among the different groups of diseases.

“Potential molecular diagnosis” was stablished with a VUS in a dominant gene potentially explaining the phenotype, two VUS in a recessive gene potentially explaining the phenotype or VUS in a X-linked gene.

“Partial molecular diagnosis” includes cases with only one P or LP variant in a recessive gene (monoallelic) potentially explaining the phenotype.

“No molecular diagnosis” was defined when no variants were reported or when either a monoallelic P, LP or VUS in recessive gene or a VUS in a dominant gene not clearly related with the clinical suspicion was detected.

For data analysis, we established different groups of diseases based on the more demanding Clinical Departments (Ophthalmology, Neurology, Cardiology, Endocrinology, Nephrology) and/or on the more demanding diseases (Retinal disorders, Hearing impairment, Skeletal dysplasia, Cystic Fibrosis, Familial hypercholesterolemia and Miscellanea group). Within the majority of them, different subgroups of diseases were established.

**Figure 1:**
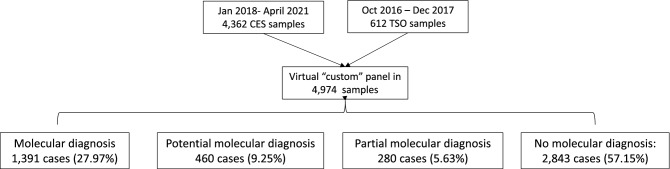
Summary and overview of the 4974 analysed cases. “Molecular diagnosis”: a P or LP variant in a dominant or X linked gene; as two P or LP, one P and one LP variant in a recessive gene, respectively, sex regardless; “Potential molecular diagnosis”: a VUS in a dominant gene potentially explaining the phenotype, two VUS in a recessive gene potentially explaining the phenotype or VUS in a X-linked gene. “Partial molecular diagnosis”: only one P or LP variant in a recessive gene (monoallelic) potentially explaining the phenotype. “No molecular diagnosis”: no variants or either a monoallelic P, LP or VUS in recessive gene or a VUS in a dominant gene not clearly related with the clinical suspicion were reported.

A summary an overview of the distribution of the 4974 analysed cases are shown in Fig. 1.

## Results

### Clinical exome cases and its distribution by groups of disease

A total of 4974 patients (2389 females, 48.03%, and 2585 males, 51.97%) were analysed. The mean age (± SD) at diagnosis was 36.1 ± 22.8 years, ranging from 0 to 96 years of age.

**Figure 2:**
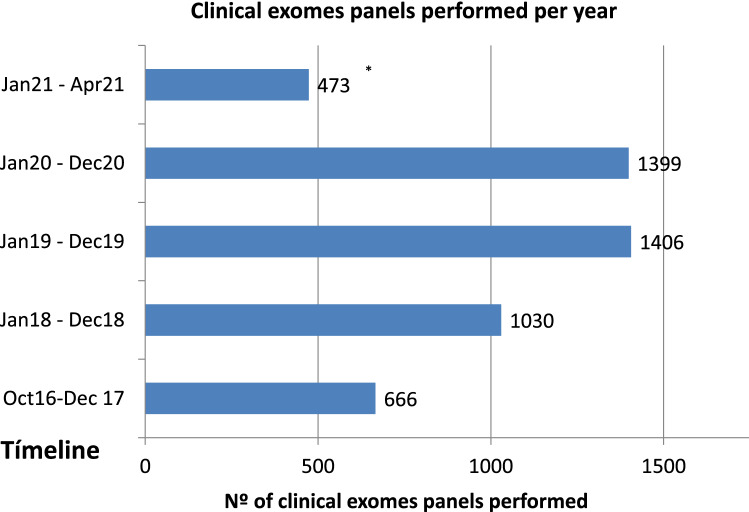
Number of clinical exomes panels performed in our laboratory per year. * Data of only 4 months.

Since the implementation of the NGS in the laboratory, we have faced a continuous and exponential growth in the number of clinical exomes requested. In the last 3 years, we approximately performed 1300–1400 clinical exomes per year (Fig. 2).

**Figure 3:**
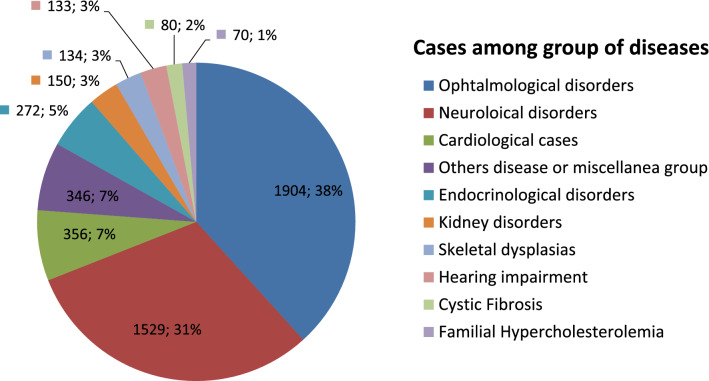
Analysed cases among groups of diseases. N = number of analysed cases; %: percentage of total (n = 4974) analysed cases.

We analysed a total of 4974 clinical exome panels. The two more frequent groups of diseases were Ophthalmological (n = 1904; 38.3%) and Neurological disorders (n = 1529; 30.74%), followed by Cardiological cases including those with clinical suspicion of Marfan syndrome, Noonan or other Rasopathies (n = 356; 7.16%). A total of 272 (5.47%) Endocrinological disorders, and 150 (3.02%) Kidney disorder cases were analysed. Skeletal dysplasias (n = 134), Hearing impairment (n = 133), Cystic Fibrosis (n = 80) and Familial Hypercholesterolemia (N = 70) corresponded to 2.69%, 2.67%, 1.61% and 1.41% of total cases, respectively. The remaining 346 cases (6.96%) corresponded to a miscellany of disorders that includes Autoinflammatory, Metabolic and Dermatology cases among others (Fig. 3).

### Diagnostic rates among established groups

A molecular and a potential molecular diagnosis was established in 27.97% (n = 1391/4974) and 9.25% (n = 460/4974) of total cases, respectively.

In 280 cases (5.63%; 280/4974), a partial molecular diagnosis was achieved. No molecular diagnosis was established in 57.16% of samples (2843/4974), including few cases with a VUS in a recessive reported gene with doubtful association with the clinical suspicion (Table 1).

**Table 1:** Molecular, potential molecular, partial molecular and no molecular diagnosis cases in the analysed cohort.

Category	N	% Total
No molecular diagnosis	2843	57.15%
Molecular diagnosis (Solved cases)	1391	27.97%
Potential molecular diagnosis	460	9.25%
Partial molecular diagnosis	280	5.63%
Total	4974	100.00%

Among the molecular and potential molecular diagnosis group in CES samples (n = 1592), a deletion or duplication was detected in 29 cases (1.8%), of which 23 (12 deletions and 11 duplications) belonged to the molecular diagnosis group (1.9%; n = 23/1191). Interestingly, the chromosomes with more detected CNVs were chromosome 15 and X (8 vs 4 cases respectively), followed by chromosomes 1 and 17 (3 cases each).

### Diagnostics rates based on group of disease

**Figure 4:**
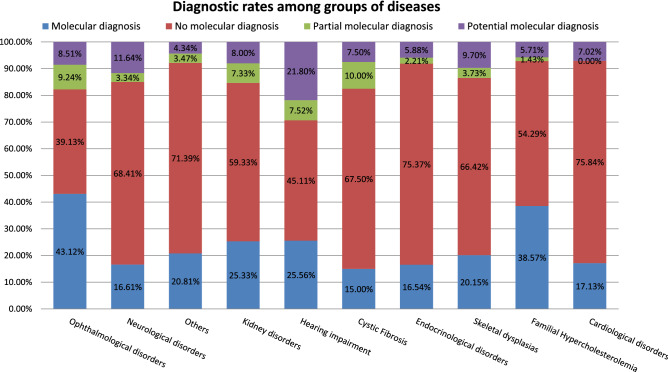
Diagnostic rates among groups of diseases. “Molecular diagnosis”: a P or LP variant in a dominant or X linked gene; as two P or LP, one P and one LP variant in a recessive gene, respectively, sex regardless; “Potential molecular diagnosis”: a VUS in a dominant gene potentially explaining the phenotype, two VUS in a recessive gene potentially explaining the phenotype or VUS in a X-linked gene. “Partial molecular diagnosis”: only one P or LP variant in a recessive gene (monoallelic) potentially explaining the phenotype. “No molecular diagnosis”: no variants or either a monoallelic P, LP or VUS in recessive gene or a VUS in a dominant gene not clearly related with the clinical suspicion were reported.

We observed that the DR was highly dependent on the indication of study and on the knowledge of the disease. The best molecular DR was achieved in cases derived from Ophthalmology (n = 821/1904; 43.12%) followed by familial hypercholesterolemia cases (n = 27/70; 38.57%) and hearing impairment (n = 31/133; 25.56%). In Nephrology (n = 38/150) and Neurological cases (n = 254/1529) we achieved a DR of 25.33% and 16.61%, respectively (Fig. 4 and Table 2).

**Table 2:** Molecular, potential molecular, partial molecular and no molecular diagnosis cases of each group of diseases. Molecular diagnosis cases were used to establish the DR among each group and subgroup of disease. Data are ordered from highest to lowest according to the number of molecular diagnosis cases. Solved cases correspond to potential and molecular diagnosis cases.

	Molecular diagnosis		Potential molecular diagnosis		Partial molecular diagnosis		No molecular diagnosis		Total
	N	%	N	%	N	%	N	%	N
**Ophthalmological disorders**	821	43.12	162	8.51	176	9.24	745	39.13	1904
Retinal dystrophy	680	47.49	132	9.22	159	11.10	461	32.19	1432
Optic Atrophy	43	24.02	4	2.23	5	2.79	127	70.95	179
Ocular malformation	76	34.55	20	9.09	12	5.45	112	50.91	220
Corneal dystrophy	22	30.14	6	8.22		0.00	45	61.64	73
**Neurological disorders**	254	16.61	178	11.64	51	3.34	1046	68.41	1529
Parkinsonism	8	11.43	5	7.14	4	5.71	53	75.71	70
Neuropathies/Paraparesia	37	18.88	29	14.80	10	5.10	120	61.22	196
Neurology others	12	20.69	2	3.45		0.00	44	75.86	58
Myopathies	34	18.89	19	10.56	5	2.78	122	67.78	180
Intellectual disability	114	16.47	76	10.98	17	2.46	485	70.09	692
Epilepsy	23	14.56	28	17.72	10	6.33	97	61.39	158
Dementia	12	11.43	7	6.67	1	0.95	85	80.95	105
Ataxia	14	20.00	12	17.14	4	5.71	40	57.14	70
Others diseases or Miscellanea group	72	20.81	15	4.34	12	3.47	247	71.39	346
**Cardiological disorders**	61	17.13	25	7.02		0.00	270	75.84	356
Rasophaties	7	20.59		0.00		0.00	27	79.41	34
Marfan syndrome	5	13.51		0.00		0.00	32	86.49	37
Cardiology	49	17.19	25	8.77		0.00	211	74.04	285
**Endocrinological disorders**	45	16.54	16	5.88	6	2.21	205	75.37	272
**Kidney disorders**	38	25.33	12	8.00	11	7.33	89	59.33	150
Nephrology others	34	27.42	11	8.87	10	8.06	69	55.65	124
Alport syndrome	4	15.38	1	3.85	1	3.85	20	76.92	26
**Hearing impairment**	34	25.56	29	21.80	10	7.52	60	45.11	133
**Familial hypercholesterolemia**	27	38.57	4	5.71	1	1.43	38	54.29	70
**Skeletal dysplasia**	27	20.15	13	9.70	5	3.73	89	66.42	134
**Cystic Fibrosis**	12	15.00	6	7.50	8	10.00	54	67.50	80
Total	1391	27.97	460	9.25	280	5.63	2843	57.16	4974

As shown in Table 2, the DR achieved is significantly different when comparing retinal dystrophy (n = 680/1432; 47.49%) to optic atrophy (n = 43/179; 24.02%) or ataxia (n = 37/196; 18.88%) or intellectual disability (114/692; 16.47%) to dementia (n = 12/105; 11.43%), among others (Table 2).

### Distribution of genes involved in molecular diagnosis

Specifically, in molecular diagnosis cases and excluding cases with deletions/duplications, genes observed 10 or more times and the five most mutated genes among the different disorders are shown in Table 3.

**Table 3:** Most frequent mutated genes in molecular diagnosis cases excluding deletions/duplications cases (n = 1368) among the different disorders and ordered by frequency. Only mutated genes observed 10 or more times or the five most mutated genes (if found more than once) among the different disorders are shown.

Group of disorders	N	%
**Ophthalmological disorders**	821	60.01
*ABCA4*	137	16.69
*USH2A*	84	10.23
*BEST1*	31	3.78
*OPA1*	29	3.53
*PRPH2*	27	3.29
*CRB1*	21	2.56
*PAX6*	19	2.31
*CNGB3*	17	2.07
*EYS*	17	2.07
*RHO*	16	1.95
*NR2E3*	15	1.83
*RPGR*	15	1.83
*PRPF31*	14	1.71
*MYO7A*	13	1.58
*RS1*	12	1.46
*CHM*	11	1.34
*CRX*	10	1.22
*PROM1*	10	1.22
*BBS1*	10	1.22
*SNRNP200*	10	1.22
*TGFBI*	10	1.22
**Neurological disorders**	235	17.18
*ANKRD11*	9	3.83
*CLCN1*	7	2.98
*NOTCH3*	6	2.55
*MECP2*	6	2.55
*KRIT1*	5	2.13
*SCN1A*	5	2.13
**Others or Miscellanea group**	72	5.26
*ATP7B*	5	6.94
*HMBS*	4	5.56
*ACVRL1*	4	5.56
*NF1*	3	4.17
*ENG*	3	4.17
**Cardiological disorders**	61	4.46
*MYBPC3*	10	16.39
*TTN*	8	13.11
*MYH7*	5	8.20
*DSP*	3	4.92
*FBN1*	3	4.92
**Endocrinological disorders**	45	3.29
*GCK*	11	24.44
*HNF1A*	6	13.33
*FGFR1*	3	6.67
*AR*	3	6.67
*PDX1*	2	4.44
*SPRY4*	2	4.44
*HNF4A*	2	4.44
**Kidney disorders**	37	2.70
*COL4A4*	10	27.03
*COL4A3*	8	21.62
*PKHD1*	2	5.41
*HNF1B*	2	5.41
**Hearing impairment**	33	2.41
*MYO7A*	5	15.15
*GJB2*	4	12.12
*MYO6*	3	9.09
*COL4A3*	2	6.06
*TECTA*	2	6.06
**Familial Hypercholesterolemia**	27	1.97
*LDLR*	25	92.59
**Skeletal dysplasia**	25	1.83
*COL2A1*	5	20.00
*COL1A1*	4	16.00
*SHOX*	2	8.00
**Cystic Fibrosis**	12	0.88
*CFTR*	12	100.00
Total	1368	100.00

## Discussion

Over the last 5 years we have implemented clinical exome sequencing as the first line test for many Mendelian disorders. This has led to a continuous and exponential growth in the number of cases analysed (Fig. 2), reflecting the increasing impact and the importance of genetics in the medical field^17^.

ES is a robust and cost-efficient diagnostic tool but, based on large cohort studies, it only provides an overall molecular diagnosis in 25–30% of cases^4,6^. In our cohort, we obtained a molecular diagnosis in 1391 patients out of the 4974 analysed cases, corresponding to an overall DR of 24.62%. Although this DR could seem lower than reported, we should consider that an important number of potential molecular cases will be likely reclassified to molecular diagnosis cases after family segregation and/or after the upgrade of the VUS, thus, increasing our DR. It has been reported that approximately 74.6% and 25.4% of VUS are further respectively reclassified to benign/likely benign and to pathogenic/likely pathogenic variants^18^. Therefore, if we consider that 70% of our potential molecular diagnosis cases (n = 460) correspond to cases with a VUS in a dominant gen, we could estimate that 17% of potential molecular cases will be reclassified to molecular cases, giving a global DR of around 29% (1469/4974), which falls into the rates previously reported by other groups.

As already described, the DR of ES is very variable depending on the analysed disorder^7^. Based on the literature, it is estimated to be approximately of 36% in developmental disorders^19,20^ but around 50% and 58% in hearing loss and retinal dystrophies, respectively^21,22^. In our cohort, we had the higher DR in ophthalmological and familiar hypercholesterolemia cases (43.12% and 38.57% respectively) compared to other disorders such as neurological cases (16.61%), cardiology (17.13%) or others (20.81%) (Table 2). However, we noticed that the DR was disorder-specific rather than dependent on the group of diseases. For instance, we observed that the DR in retinal dystrophies (47.49%) was almost twice than in optic atrophy (24.02%), and in neuropathies/paraparesias was 18.88% while it was 11.43% in dementia cases. Probably, these differences are a direct consequence of the general knowledge about the genes associated to the disease, of our capacity to detect the causal gene and of the probability of a non-Mendelian aetiology of the disorder. The latter is reflected for example in the DR of dementia (11.43%) or parkinsonism (11.43%) vs retinal dystrophy (49.49%) or hearing impairment (25.56%). It is important to point out that we are not evaluating digenic inheritance or dual/multiple diagnoses, being those one of the main limitations of our study.

CNVs have been associated with a broad range of pathological conditions, accounting approximately for 13% of the human genome alterations^23^. They could not be detected in our TSO analysed samples; however, at least in four TSO cases with high clinical suspicion and one allele detected in a disease-related gene, a definitive molecular diagnosis was established thanks to further MLPA analysis. Within the total 4362 CES samples analysed, we detected a deletion/duplication in 29 cases, of which 23 CNVs were detected within the molecular diagnosis group of CES samples, corresponding to a 1.9% (23/1191). Probably, we missed others due to the limitations of the CNVs detection by ES, which depends on the capture regions, the homogeneity and the coverage obtained for each sample.

The NGS diagnosis success strongly relies on the analysis and the strategy used for gene selection and variant filtering^24^. The pipeline used during the analysis is also essential to improve diagnosis, as reflected in our capacity to increase the DR of previously unsolved cases in 2.5%, 3.2% and 4.4% of cancer, cardiovascular and retinal dystrophy cases respectively, as recently reported by our group^25^. In addition, reanalysis of ES data represents a powerful approach to identify additional diagnosis and improve patient care^26–28^. According to recent studies, exome reanalysis improves the DR in approximately 12%, with reported increases ranging from 5 to 26%^29^. It is known that this increased DR is mainly due to the discovery of new disease-genes, the use of better bioinformatics tools, such as large databases and improved pipelines that help in the reclassification of variants, as previously demonstrated by our group^30,31^, and the availability of additional phenotype data, among others^16^. In this line, prioritized features and genes using Human Phenotype Ontology terminology have been proposed as a helping diagnostic tool^32,33^. In addition, good communication with the clinicians ordering tests and an continuous genomic data sharing, is essential for the discovery of new genes -as already demonstrated with the GeneMatcher tool^34–36^-and for a better interpretation of variants that will definitely improve our diagnosis process^3^. Given the evolving nature of the genomic field, it is also essential to implement the exome reanalysis as part of routine clinical practice^25,29,37^.

Big efforts are still needed to improve our genetic DR^14^. Although ES is gradually being optimized, it has limitations and in some cases generates laboratory’s errors difficult to avoid^38^. In this situation, whole genome sequencing (WGS) is becoming an attractive alternative as it is presumably able to detect more potential pathogenic variants than ES^39–41^. Thanks to its greater breadth of coverage, genome sequencing (GS) is better than ES to detect single nucleotide variants and CNVs, also allowing the detection of variants in non-coding regions and chromosome rearrangements, all of which have been demonstrated to be involved in human disease^3,42,43^. It is not casual that if we exclude cystic fibrosis cases due to its high prevalence in the general population, the higher partial molecular diagnosis is achieved in ophthalmological (9.2%), kidney (7.33%) and hearing impairment (7.52%) cases, in which the highest DR is reported in the literature. Undoubtedly, the analyses of non-coding regions or improved CNVs would help us to detect the second missed allele, increasing our global DR with the corresponding benefit for the patients.

## Conclusions

Here, we present the DR of the CES we routinely perform in our clinical practice. Probably, the case selection, the continuous communication with clinicians and the expertise of our genetics team, make the difference in favour of our global estimated DR of 29%. Nevertheless, this overall DR is still low and direct efforts on the analysis and interpretation of NGS are required. Large databases, improved pipelines, data sharing and further investigations for a deeper understanding of ES, GS and other focused genetics techniques are needed in order to improve our global DR in the benefit of patients.

## Data Availability

All data generated or analysed during this study are included in this published article.
